# Canine *Angiostrongylus vasorum*-Induced Early Innate Immune Reactions Based on NETs Formation and Canine Vascular Endothelial Cell Activation In Vitro

**DOI:** 10.3390/biology10050427

**Published:** 2021-05-12

**Authors:** Daniela Grob, Iván Conejeros, Sara López-Osorio, Zahady D. Velásquez, Lisa Segeritz, Ulrich Gärtner, Roland Schaper, Carlos Hermosilla, Anja Taubert

**Affiliations:** 1 Institute for Parasitology, Justus Liebig University Giessen, 35392 Giessen, Germany; Ivan.Conejeros@vetmed.uni-giessen.de (I.C.); sara.lopezo@udea.edu.co (S.L.-O.); zahady.velasquez@vetmed.uni-giessen.de (Z.D.V.); Lisa.C.Segeritz@vetmed.uni-giessen.de (L.S.); Carlos.R.Hermosilla@vetmed.uni-giessen.de (C.H.); Anja.Taubert@vetmed.uni-giessen.de (A.T.); 2 Grupo de Investigación CIBAV, Universidad de Antioquia UdeA, Medellín 050034, Colombia; 3 Institute of Anatomy and Cell Biology, Justus Liebig University Giessen, 35392 Giessen, Germany; ulrich.gaertner@anatomie.med.uni-giessen.de; 4 Elanco Animal Health, 40789 Monheim, Germany; roland.schaper@elancoah.com

**Keywords:** *Angiostrongylus vasorum*, canine PMN, NETs formation, primary canine aortic endothelial cells, adhesion molecules

## Abstract

**Simple Summary:**

*Angiostrongylus vasorum* is a cardiopulmonary nematode that affects canids, residing in the pulmonary artery and right atrium/ventricle. Due to its location, the parasite will have a close interaction with the different components of the innate immune system, including endothelial cells and polymorphonuclear neutrophils (PMN). Here we evaluated the expression of adhesion molecules of canine aortic endothelial cells (CAEC), and NETs formation by co-culture of freshly isolated canine PMN with *A. vasorum* L3. Overall, we found distinct inter-donor variations in adhesion molecule expression among CAEC isolates. Additionally, PMN and *A. vasorum* co-culture induced NETs release without affecting larval viability.

**Abstract:**

Due to its localization in the canine blood stream, *Angiostrongylus vasorum* is exposed to circulating polymorphonuclear neutrophils (PMN) and the endothelial cells of vessels. NETs release of canine PMN exposed to *A. vasorum* infective stages (third stage larvae, L3) and early pro-inflammatory immune reactions of primary canine aortic endothelial cells (CAEC) stimulated with *A. vasorum* L3-derived soluble antigens (*Av*Ag) were analyzed. Expression profiles of the pro-inflammatory adhesion molecules ICAM-1, VCAM-1, P-selectin and E-selectin were analyzed in *Av*Ag-stimulated CAEC. Immunofluorescence analyses demonstrated that motile *A. vasorum* L3 triggered different NETs phenotypes, with spread NETs (*spr*NETs) as the most abundant. Scanning electron microscopy confirmed that the co-culture of canine PMN with *A. vasorum* L3 resulted in significant larval entanglement. Distinct inter-donor variations of P-selectin, E-selectin, ICAM-1 and VCAM-1 gene transcription and protein expression were observed in CAEC isolates which might contribute to the high individual variability of pathological findings in severe canine angiostrongylosis. Even though canine NETs did not result in larval killing, the entanglement of L3 might facilitate further leukocyte attraction to their surface. Since NETs have already been documented as involved in both thrombosis and endothelium damage events, we speculate that *A. vasorum*-triggered NETs might play a critical role in disease outcome in vivo.

## 1. Introduction

*Angiostrongylus vasorum* is a metastrongyloid nematode causing cardiopulmonary disorders in domestic dogs [1,2,3]. In vivo, *A. vasorum* resides in pulmonary arteries and the right side of the heart of domestic dogs and other carnivores [4,5,6]. The life cycle is heteroxenous and various terrestrial gastropod genera act as obligate intermediate hosts. They become infected by the consumption of first-stage larvae (L1) released into the environment by feces of *A. vasorum*-infected canids [7,8,9]. In gastropods, *A. vasorum* L1 develop into infective third-stage larvae (L3) which are ingested by definitive hosts to achieve the life cycle [10]. Over the last decades, canine angiostrongylosis has geographically spread into previously non-endemic areas and life-threatening cases have increasingly been reported [11,12,13,14]. Nowadays, canine angiostrongylosis is considered an emerging disease not only in Europe but also in North and South America [9,13,15,16,17]. *A. vasorum*-infected dogs show a wide range of clinical signs from mild coughing to neurological disorders, along with the presence of respiratory symptoms, coagulopathies (e.g., vascular thrombosis, diathesis, hemorrhages), gastrointestinal disorders, leukophilia and hypercalcemia representing common findings [18,19,20].

Based on its life cycle and migratory routes in the final host, direct contacts of different *A. vasorum* stages (i.e., L3, pre-adults and adults) with both canine polymorphonuclear neutrophils (PMN) and vascular endothelial cells will indeed occur in vivo. PMN are the most abundant leukocytes and represent the first line of defense in mammalian hosts [21,22,23]. PMN are recruited immediately after pathogen invasion and exhibit different effector mechanisms: degranulation of immunomodulatory molecules, generation of reactive oxygen species (ROS), phagocytosis and NETosis (release of neutrophil extracellular traps, NETs). NETs are delicate extracellular structures formed by decondensed chromatin, mainly via PAD4-mediated citrullination, and adorned with antimicrobial components, such as myeloperoxidase (MPO), neutrophil elastase (NE), lactoferrin, calprotein, LL37, pentraxin, proteinase 3 or cathepsin G [21,24,25]. Meanwhile, different NET phenotypes were reported, including diffuse (*diff*NETs), spread (*spr*NETs), aggregated (*agg*NETs), cell free and anchored NETs [26,27,28]. *diff*NETs consist of globular and compact forms with sizes of 15–20 nm diameter whilst *spr*NETs are smooth and elongated web-like structures with extremely thin fibers of 15–17 nm diameter [29,30]. *agg*NETs are large conglomerates with sizes >50 μm in diameter and released by a high number of PMN undergoing NETosis [29,30,31,32].

So far, canine PMN have been shown to cast NETs in response to LPS, PMA [33], sodium arsenic [34], platelet activating factor (PAF) [35], *Neospora caninum* [36] and the heartworm *Dirofilaria immitis* [30]. Even though NETs in general were proven effective against nematode stages of *Haemonchus contortus, Strongyloides stercoralis, Ostertagia ostertagi* and *Brugia malayi* [30,37,38,39,40], nothing is currently known on canine NETosis against *A. vasorum*. So far, only one study has reported on *A. vasorum-*triggered phagocyte-derived extracellular traps (ETs) formation in the gastropod immune system [8].

Endothelial cells are highly immunoreactive and rapidly produce a broad range of molecules (e.g., adhesion molecules, cytokines, chemokines) upon activation, thereby triggering pro-inflammatory responses [41,42]. Interestingly, tight interactions between activated endothelium and NETs, but also adverse effects of NETs on endothelial integrity, were reported [14,43]. As indication of chronic inflammation, leukocytosis and neutrophilia have already been described in *A. vasorum*-infected animals [20]. Likewise, immune-mediated inflammation and damage of lung vasculature were also reported for *A. vasorum* infections in dogs [20,44]. However, detailed analysis of endothelial activation during *A. vasorum* infection in dogs is scarce. Interestingly, the closely related nematode *Angiostrongylus cantonensis* induces an increase in the blood–brain barrier via metalloproteinase 9 upregulation [45], thereby suggesting endothelial cell activation. In this context, related to the case of another heartworm that affect canids, *D. immitis*, it is well-known that its presence in the blood stream of the host causes endothelial cell activation and inflammation, a situation demonstrated in vivo and in vitro [46], situation attributed to progressive pulmonary endarteritis and muscular hypertrophy of arteriole walls [44,47]. Activated endothelial cells not only trigger leukocyte recruitment and adhesion but also secrete von Willebrand factor (vWF), a large multifunctional glycoprotein with strong adhesive properties mediating adhesion of platelets at the site of the vascular damage [48,49,50,51]. As an indirect evidence of infection-induced endothelial cell activation, increased vWF concentrations were found in 32% of *A. vasorum*-infected dogs suffering bleeding disorders [12].

To our best knowledge, we here show for the first time that motile axenic *A. vasorum* L3 and soluble *A. vasorum* L3 antigens (*Av*Ag) induce both significant NET formation and canine endothelial cell activation, and suggest that these reactions may contribute to individual disease outcome.

## 2. Materials and Methods

### 2.1. Gastropod Maintenance and Isolation of Axenic Angiostrongylus vasorum Third-Stage Larvae (L3)

Terrestrial leopard slugs (*Limax maximus*) were bred and maintained in fully-automated climate incubators (model ECP01E; Snijders Scientific B.V. Tilburg, the Netherlands) according to [8]. Briefly, breeding and maintenance was performed under controlled conditions: 50% humidity, 10 h of dark/10 h of illumination corresponding to circadian cycles, plus 2 h for dusk and dawn each, temperature ranging from 10 to 16 °C (night/day). *L. maximus* were kept on wet paper towels in plastic boxes equipped with Petri dishes for food supply and a plastic dim housing area (Techniplast, Hohenpeissenberg, Germany) for slug retreat.

*A. vasorum* first-stage larvae (L1) were isolated via the Baermann funnel technique from feces of experimentally infected dogs (kindly provided by the Institute of Parasitology, University of Veterinary Medicine of Hannover, Hannover, Germany) as described elsewhere [8]. Approximately 10 mL of sediment containing migrated L1 were collected and pelleted (800× *g*, 5 min, 20 °C). The supernatant was discarded and larval numbers were determined microscopically (Olympus CX41). Three leopard slugs (*L. maximus*) were orally infected with 2000 vital larvae each and according to [8,17].

### 2.2. Isolation of Canine PMN

Blood samples were collected from healthy adult Beagle dogs (*n =* 7; Marshall BioResources, kept at Elanco Animal Health, Monheim, Germany) and used for canine PMN isolation. Heparinized blood was diluted in an equal volume of sterile PBS with 0.02% EDTA (Sigma-Aldrich, Darmstadt, Germany) and placed on Biocoll Separating Solution^®^ (Biochrom AG, Berlin, Germany). The samples were centrifuged at 800× *g* for 45 min at RT. The cell pellet was gently re-suspended, diluted in 27 mL of distillated water and shaken for 20 s to lyse erythrocytes according to [30]. Then, osmolarity was adjusted by adding 3 mL of 10× Hanks Salt Solution (HBSS, Biochrom AG, Berlin, Germany). Canine PMN were washed twice (400× *g,* 10 min, RT), re-suspended in sterile RPMI 1640 medium (Sigma-Aldrich, Darmstadt, Germany), counted in a Neubauer hemocytometer chamber and incubated at 37 °C with 5% CO_2_ for 30 min before experimental use.

### 2.3. Scanning Electron Microscopy (SEM) Analysis

Canine PMN were co-cultured with axenic vital *A. vasorum* L3 (6 larvae/sample) on poly-_L_-lysine (Sigma-Aldrich, Darmstadt, Germany) pre-coated coverslips (60 min, RT). After incubation, the samples were fixed in HEPES solution (Sigma-Aldrich; 0.3 M, pH 7.35) containing 1.5% paraformaldehyde and glutaraldehyde (both Merck, Darmstadt, Germany) (60 min, RT), post-fixed in 1% osmium tetroxide (Merck, Darmstadt, Germany), washed in sterile distilled water, dehydrated, critical point dried by CO_2_ treatment and sprayed with gold. Thereafter, samples were analyzed with a Philips XL30^®^ scanning electron microscope at the Institute of Anatomy and Cell Biology, Justus Liebig University Giessen, Giessen, Germany.

### 2.4. NET Visualization by Immunofluorescence

Canine PMN (4.2 × 10^5^/400 µL) were seeded on 15 mm diameter poly-_L_-lysine treated coverslip glasses (Nunc, Schwerte, Germany) and placed in a 12 well plate (Greiner, Frickenhausen, Germany) in sterile RPMI 1640 medium (without phenol red, supplemented with 1% penicillin/streptomycin, Sigma-Aldrich, Darmstadt, Germany). Co-culture of axenic *A. vasorum* L3 with canine PMN was performed for 90 min (2 × 10^5^ PMN were stimulated with 10 larvae/well, 37 °C, 5% CO_2_). Thereafter, samples were fixed in 4% paraformaldehyde (Merck, Darmstadt, Germany) and kept at 4 °C until further analysis. Canine NET structures were visualized by staining extracellular DNA with DAPI (4′,6-diamidino-2-phenylindole, Thermo Fisher Scientific, Langenaselbold, Germany). For the detection of histones and NE decorating NETs structures, the following primary antibodies were used: anti-global histone (H1, H2A/H2B, H3, H4) (clone H11-4, 1:500; Merck Millipore cat #MAB3422) and anti-NE (cat # Ab68672, 1:500, Abcam, Berlin, Germany). Briefly, samples were washed with sterile PBS, blocked in 3% bovine serum albumin (BSA) (Sigma-Aldrich; 60 min, RT) + 0.3% Triton X-100 (Thermo Fischer Scientific, Langenselbold, Germany) for 60 min at RT and then incubated with respective primary antibodies for 3 h at RT. Then, samples were washed three times with sterile PBS and incubated in secondary antibody solutions (Alexa 594 goat anti-mouse IgG H&L #A11005, Alexa 488 goat anti-rabbit IgG #A11008, 1:500, RT). Finally, samples were washed three times with sterile PBS and mounted upside-down with Fluoromount G^®^ with DAPI (Thermo Fischer Scientific). Visualization of NETs was achieved using an inverted IX81 fluorescence microscope equipped with an XM10 digital camera (both Olympus, Hamburg, Germany). Five random pictures were taken from each experimental condition to analyze the presence of NETs and NET phenotypes.

### 2.5. Assessment of Different NET Phenotypes

NET phenotypes were quantified by immunofluorescence microscopy as previously described elsewhere [27]. Therefore, five randomly taken pictures were analyzed by manual counting, based on morphological and morphometric characteristics for each phenotype of canine NETs, as previously described [8,30].

### 2.6. Nuclear Decondensation-Based Quantification Using DANA Software

To further confirm parasite-induced NET induction and to evaluate effects within the dynamic NETotic process, we additionally analyzed *A. vasorum* L3-triggered nuclear area expansion (NAE) in canine PMN. Nuclear expansion-based quantification of NETs relied on the method described by [25] and the software DANA I and DANA II was applied according to the developer’s recommendations. In brief, freshly isolated canine PMN (*n* = 7 donors, 2 × 10^5^ PMN/well) were left in plain medium (RPMI 1640, Sigma-Aldrich, Darmstadt, Germany) for 30 min and then exposed to 10 L3/well for 90 min. After incubation, samples were fixed using 2% paraformaldehyde (Merck, Darmstadt, Germany) and stained with DAPI (Thermo Fischer Scientific, Langenselbold, Germany) for 30 min RT. Five microscopic images were randomly taken for each condition using an inverted microscope (Olympus IX 81), having a total of 203 PMN in the control and 178 in the stimulated group. Nuclear areas of PMN were analyzed using DANA software. 

### 2.7. Isolation of Primary Canine Aortic Endothelial Cells (CAEC)

Four aortic arteries from four adult healthy male Beagle dogs were donated from Elanco Animal Health, Monheim, Germany. Arteries were kept at 4 °C in sterile 0.9% HBSS-HEPES buffer (pH 7.4; Gibco) supplemented with 1% penicillin (500 U/mL; Sigma-Aldrich) and streptomycin (500 µg/mL; Sigma-Aldrich, Darmstadt, Germany). For isolation of aortic endothelial cells, 0.025% collagenase type II (Worthington Biochemical Corporation, Lakewood, NJ, USA) was infused into the vessel lumen, the aorta was ligated with clamps and incubated for 20 min at 37 °C in 5% CO_2_ atmosphere. After gently massaging aortas, infused collagenase II-cell suspension was collected and immediately supplemented with 1 mL sterile fetal calf serum (FCS; Gibco, Langenselbold, Germany) to inactivate collagenase II. After two washing steps (400× *g*, 10 min, 4 °C), the cells were suspended in endothelium cell growth medium (ECGM; PromoCell, Heidelberg, Germany), plated in 25 cm^2^ plastic culture flasks (Nunc, Roskilde, Denmark) and kept at 37 °C in 5% CO_2_ atmosphere until reaching confluent cell layers. Culture medium was changed every 2–3 days.

### 2.8. Preparation of Angiostrongylus vasorum L3 Soluble Antigen (AvAg)

Twenty *A. vasorum* L3 were used for soluble antigen preparation (*Av*Ag). Therefore, larval stages were frozen in liquid nitrogen and thereafter grounded in 300 µL sterile phosphate-buffered saline (PBS; 1×) in a previously UV-sterilized and cooled mortar (−80 °C for 1 h). The resulting suspension was sonicated in an ice bath with a Sonorex Super RK31^®^ bath-type sonicator (Bandelin, five cycles of 15 s) and centrifuged (10,000× *g*, 20 min, 4 °C). Final protein concentration of PBS-soluble supernatants was estimated via Coomassie Plus (Bradford, UK) Assay Kit^®^ (Thermo Scientific). *Av*Ag was stored at −20 °C until further use.

### 2.9. Total RNA Isolation and qRT-PCR

CAEC were seeded in 6-well plastic plates (Greiner, Frickenhausen, Germany) until confluence (37 °C and 5% CO_2_). Thereafter, CAEC monolayers were exposed to 1 ng/mL of soluble *Av*Ag and incubated at 37 °C with 5% CO_2_. At 3, 6, 12 and 24 h post stimulation, total RNA was harvested by applying RTL lysis buffer (Qiagen, Hilden, Germany) directly on the well. RNA isolation was performed with RNeasy kit (Qiagen, Hilden, Germany) according to manufacturer instructions and followed by a DNAse (Thermo Scientific, Langenselbold, Germany) digestion (37 °C, 30 min) to remove genomic contamination. DNAse was then inactivated by heating (65 °C, 10 min). Efficiency of DNA digestion was confirmed by including no-RT-controls in each qRT-PCR experiment. In total, 1 µg of DNAse-treated total RNA was reversely transcribed with SuperScript IV enzyme (Thermo Scientific, Langenselbold, Germany), according to manufacturer instructions. cDNA synthesis was performed for 10 min at 23 °C, then 10 min at 50 °C. The enzyme was then inactivated by heating (80 °C) for 10 min.

Probes were labelled at the 5′-end with the reporter dye FAM (6-carboxyfluorescein) and at the 3′ –end with the quencher dye TAMRA (6-carboxytetramethyl-rhodamine) (refer to Table 1). PCR amplification was performed in an automated fluorometer Rotor-GeneQ cycler (Qiagen, Hilden, Germany) using a 96-well optical plates (Greiner, Frickenhausen, Germany). Samples were analyzed in duplicate. For PCR, 2 µL cDNA (corresponding to 25 ng total RNA) were used in a 10 µl PCR reaction mixture containing 5 µL PerfeCTa FastMix II (QuantaBio, Beverly, MA, USA), 400 nM of each primer and 200 nM probe. Amplification conditions were the same for all targets assayed: one cycle at 95 °C for 5 min, one cycle at 94 °C for 15 min and one cycle at 60 °C for 60 min. Semiquantitative analyses used comparative C_t_ method (ΔΔC_t_ method, [52] and reported as *n*-fold differences in comparison to one of the samples arbitrarily chosen as calibrator). Canine ribosomal protein L32 (RPL32), ribosomal protein S19 (RPS19) and hypoxanthine phosphoribosyl-transferase (HPRT) genes were used as housekeeper genes. TNFα (10 ng/mL for 24 h, Serotec) was used a positive control.

### 2.10. Protein Isolation and Western Blot Analyses

CAEC were seeded in 6-well plastic plates (Greiner, Frickenhausen, Germany) until confluence at 37 °C and 5% CO_2_ atmosphere. Then, CAEC layers where stimulated with 1 ng/mL *Av*Ag for 3, 6, 12 and 24 h (37 °C, 5% CO_2_). Thereafter, samples were subjected to protein isolation: proteins from CAEC were extracted by cell sonication (20 s, 5 cycles) in RIPA buffer (50 mM Tris-HCl, pH 7.4; 1% NP-40; 0.5% Na-deoxycholate; 0.1% SDS; 150 mM NaCl; 2 mM EDTA; 50 mM NaF, all Roth) supplemented with a protease inhibitor cocktail (1:200, Sigma-Aldrich). Cell homogenates were centrifuged (10,000× *g*, 10 min, 4 °C) to sediment intact cells and nuclei. The RIPA buffer-soluble protein content of supernatants was quantified via Coomassie Plus (Bradford) Assay Kit^®^ (Thermo Scientific, Langenselbold, Germany) following the manufacturer’s instructions.

For immunoblotting, samples were supplemented with 6 M urea protein loading buffer. After boiling (95 °C) for 5 min, proteins (20 µg/slot) were separated in 12% or 15% polyacrylamide gels via electrophoresis (100 V, 1.5 h; *tetra* system, BioRad, Dreieich, Germany). Proteins were then transferred to polyvinylidene difluoride (PVDF) membranes (Millipore) (300 mA, 2 h at 4 °C). Blots were blocked in 3% BSA in TBS (50 mM Tris-Cl, pH 7.6; 150 mM NaCl containing 0.1% Tween (blocking solution); Sigma-Aldrich (Darmstadt, Germany) for 1 h at RT and then incubated overnight at 4 °C in primary antibodies against vinculin (1:1000, #sc-73,614, Santa Cruz), E-selectin (1:500, #TA318934, OriGene, Herford, Germany), P-selectin (1:500, #TA318936, OriGene, Herford, Germany) and VCAM-1 (1:500, #TA502391, OriGene, Herford, Germany) diluted in blocking solution. Vinculin detection was used as loading control for sample normalization. Following three washings in TBS-Tween 0.1% buffer (Sigma-Aldrich, Darmstadt, Germany), blots were incubated for 30 min at RT with secondary antibodies (goat anti-mouse IgG peroxidase-conjugated (1:40,000, #31,430, Pierce, Langenselbold, Germany); goat anti-rabbit IgG peroxidase-conjugated (1:40,000, #31,460, Pierce, Langenselbold, Germany) diluted in blocking solution. After three further washings in TBS-Tween 0.1% buffer, signal detection was accomplished by an enhanced chemo-luminescence detection system (ECL^®^ plus kit, GE Healthcare) and recorded using a ChemoCam^®^ Imager (Intas Science Imaging, Göttingen, Germany). Protein sizes were controlled by a protein ladder (PageRuler Plus^®^ Prestained Protein Ladder ~10–250 kDa, Thermo Fisher Scientific, Langenselbold, Germany). Protein band intensities were quantified by Image J^®^ (NIH), Fiji Gel Analyzer plugin.

### 2.11. Statistical Analysis

For all analyses except for NAE analysis in which ANOVA was applied, statistical significance was defined by a *p* value ≤ 0.05 determined by non-parametric analyses: Mann-Whitney test when two experimental conditions were compared and Kruskal-Wallis test followed by Dunn’s post-hoc test for multiple comparisons. All graphs (mean ± SD) and statistical analyses were performed using Graph Pad^®^ software (v.7.03).

## 3. Results

### 3.1. Angiostrongylus vasorum L3 Trigger NET Formation in Canine PMN and Led to Differential NET Phenotype Formation

**Figure 1:**
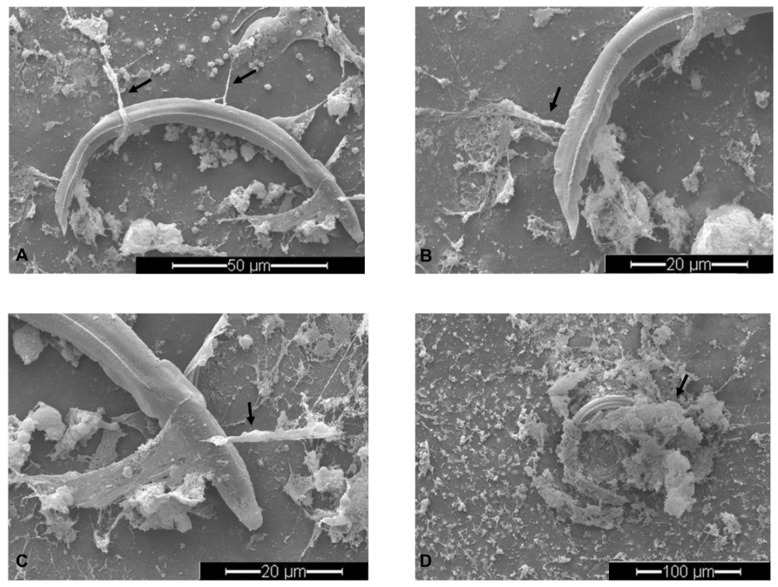
*Angiostrongylus vasorum* L3-induced neutrophil extracellular traps (NETs) analyzed via scanning electron microscopy (SEM) analysis. Fine spread NETs (*spr*NETs) entrapping the L3 (**A**–**C**) and robust aggregated NETs (*agg*NETs) (**D**) were the most predominant phenotypes of NETs. Arrows point to NET-like delicate PMN-derived structures in co-culture assays.

SEM analysis demonstrated that co-culture of canine PMN with live *A. vasorum* L3 induced the formation of NET-like thick and fine DNA fibers originating from dead PMN attached to L3 stages (Figure 1, arrows). As such, robust PMN-derived structures contacted and firmly entrapped *A. vasorum* L3. However, not all canine PMN participated in NETs release after exposure to highly motile larval stages. As such, a large number of PMN did not show morphological changes and a small number of non-NETotic PMN were also found to be firmly attached to the parasite’s cuticle (Figure 1A–C).

**Figure 2:**
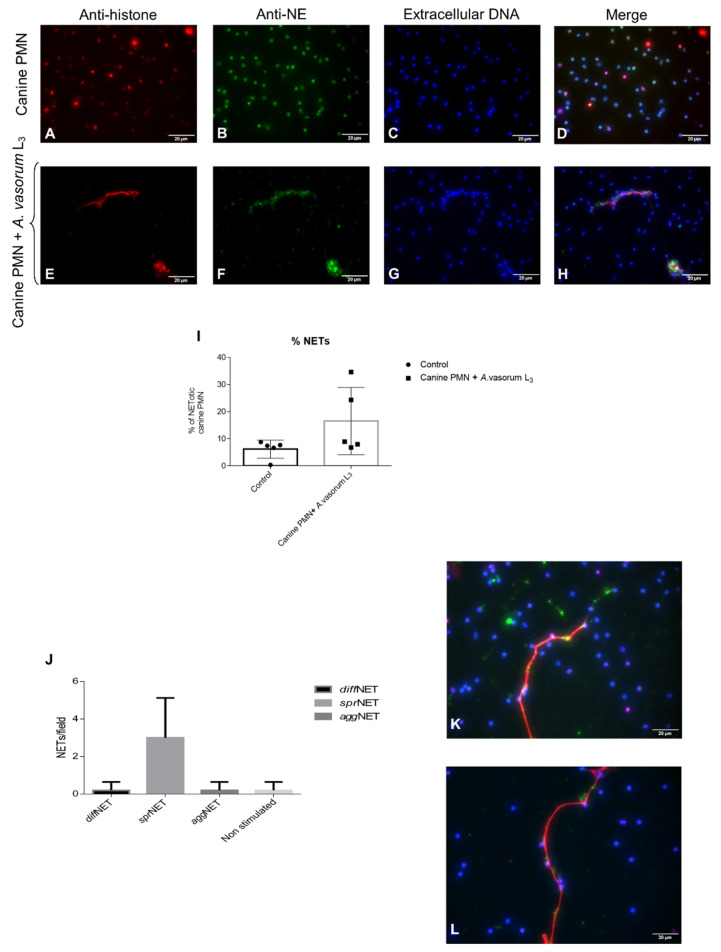
Immunofluorescence analyses of *Angiostrongylus vasorum* L3-induced neutrophil extracellular trap (NET) formation. Co-localization analyses on extracellular DNA, histones, and neutrophil elastase were performed. Presence of extracellular DNA (**C**,**G**; blue), anti-histone (**A**,**E**; red) and anti-NE (**B**,**F**; green) was confirmed. (**D**,**H**) depicts the merging of the three channels. (**I**) reveals the percentage of *A. vasorum* L3-triggered NETosis. (**J**–**L**) demonstrate the presence of spread NETs (*spr*NETs).

To prove that these *A. vasorum* L3-triggered structures were, indeed, NETs, immunofluorescence analyses were performed to detect classical NETs components. DAPI staining confirmed the DNA nature of extracellular NET-like structures extruding from ruptured canine PMN after exposure to *A. vasorum* L3 larvae (Figure 2D,H). Additionally, co-localization analyses revealed the simultaneous presence of NE (Figure 2B–F) and global histones (Figure 2A–E) in DNA-positive (Figure 2C–G) canine NETs (Figure 2D–H).

When considering different NET phenotypes, vital *A. vasorum* L3 mainly triggered the formation of *spr*NETs (Figure 2J), consisting of smooth and elongated web-like structures of decondensed chromatin and antimicrobial proteins with a diameter of 15–17 µm (Figure 2K–L). To a lesser extent, the formation of *diff*NET- and *agg*NET-phenotypes was induced. Whilst the former were composed of extracellular chromatin complexes covering a larger area and decorated with antimicrobial proteins of globular and compact form, *agg*NETs were much larger in size (≥ 50 µm) and originated from groups of NETotic PMN. Overall, mainly *agg*NETs appeared rigid enough to immobilize these large and highly motile larval stages.

**Figure 3:**
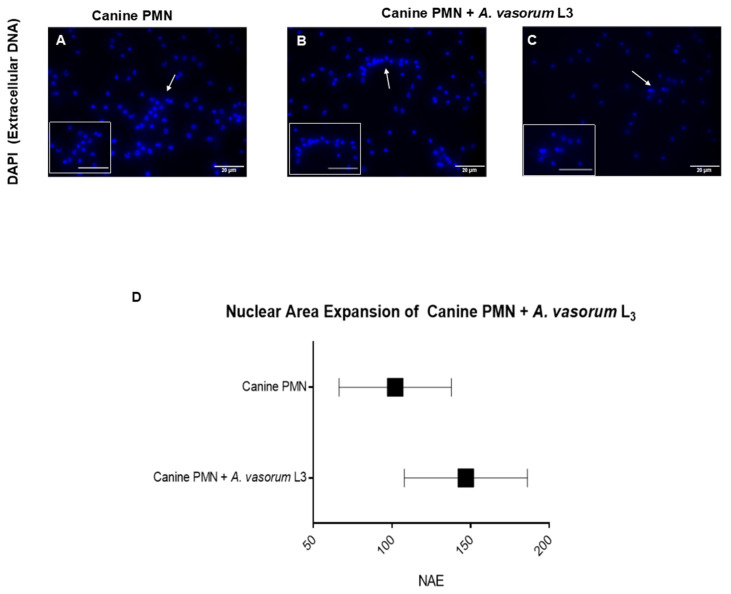
Nuclear expansion (NAE)-based quantification of *A. vasorum* L3-triggered NETs. Canine PMN were incubated in cell medium alone or exposed to *A. vasorum* L3. (**A**) depicts canine PMN alone; (**B**,**C**) illustrates co-cultivation of canine PMN + *A. vasorum*. Box placed on the left bottom depicts an isolated magnification of canine PMN alone and canine PMN co-cultured with axenic *A. vasorum* L3. NAE was analyzed by ImageJ^®^ and DANA software. (**D**) shows the nuclear area increase of NETotic cells after co-cultures.

Observer-based estimation of cell numbers of performing NETs revealed that 13.2% of PMN released NETs when confronted with vital *A. vasorum* L3 (Figure 2I). In comparison, only 5.3% of the total PMN population reacted in this manner in control conditions (unstimulated PMN). Figure 3A depicts the normal structure of a canine PMN and its nucleus. When canine PMN were exposed to *A. vasorum* L3 stages, we observed a mean expansion of the NAE of 102.1 ± 35.64 µm^2^ in control cells, whilst in parasite-encountering cells this area increased to 146.9 ± 39.11 µm^2^ (controls vs. *A. vasorum*: *p* = 0.02; Figure 3B–D).

### 3.2. AvAg Induces Canine Endothelial Cell Activation and Donor-Dependent Adhesion Molecule Expression

**Figure 4:**
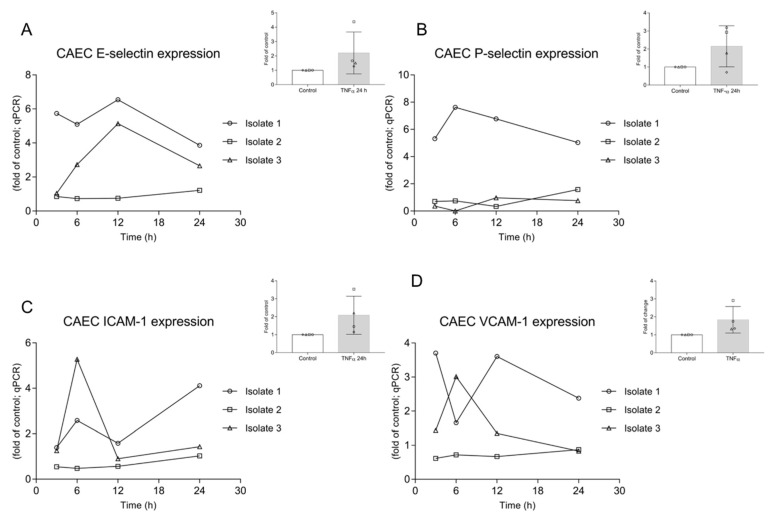
Adhesion molecule gene transcription in *A. vasorum* antigen (*Av*Ag)-stimulated canine endothelial cells. CAEC were stimulated with soluble *A. vasorum* L3 antigen (*Av*Ag; 1 ng/mL) and after 3, 6, 12 and 24 h of incubation total RNA was isolated, reverse transcribed and assayed for E-selectin (**A**), P-selectin (**B**), ICAM-1 (**C**) and VCAM-1 (**D**) gene transcription via qPCR. Data are expressed as *n*-fold of controls (non-stimulated CAEC). TNF-α stimulation was used as positive control.

**Figure 5:**
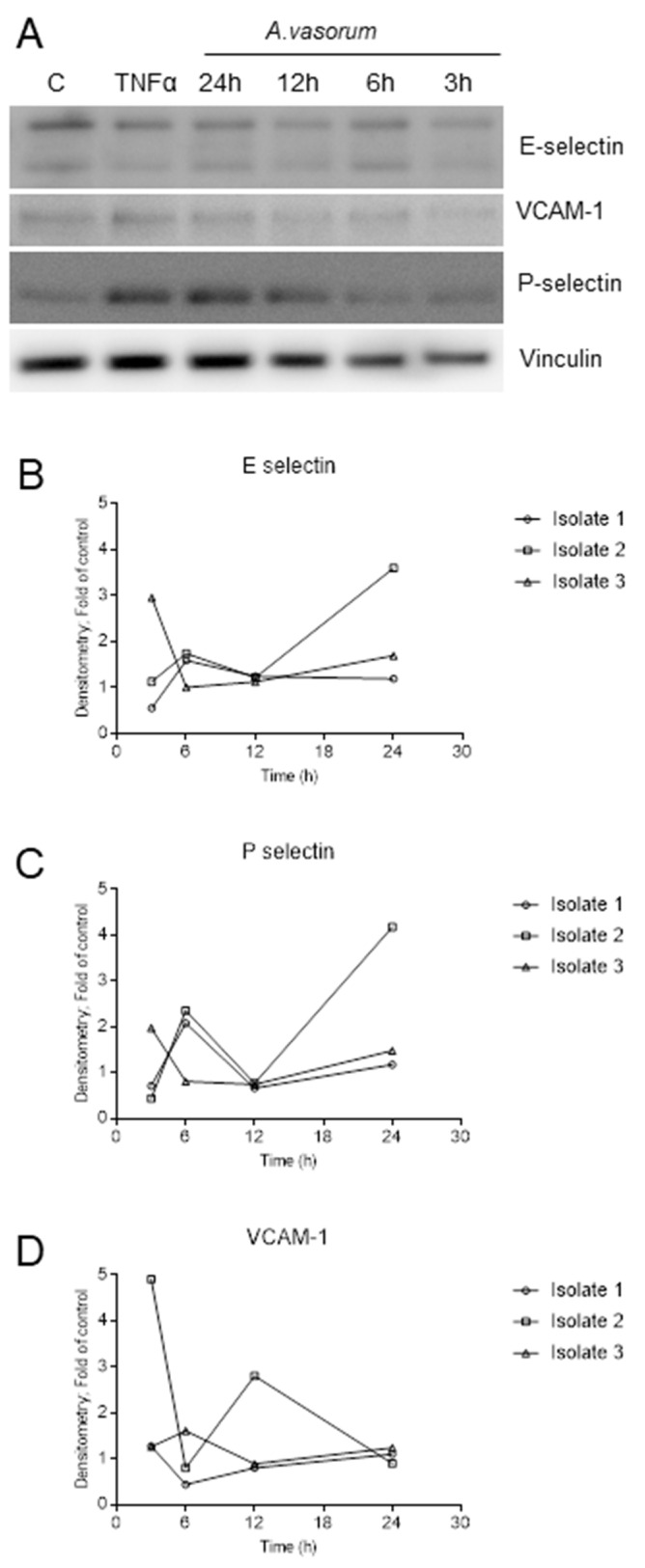
Adhesion molecule protein expression in *A. vasorum* antigen (*Av*Ag)-stimulated canine endothelial cells. CAEC cells were stimulated with *A. vasorum* L3 antigen (*Av*Ag; 1 ng/mL) and after 3, 6, 12 and 24 h of incubation, total protein was extracted. Non-stimulated CAEC served as negative controls. The expression of E-selectin (**B**), P-selectin (**C**) and VCAM-1 (**D**) was studied by Western blotting (**A**); representative illustration of one CAEC isolate and densitometric analysis of protein bands. TNF-α was used as positive control of CAEC stimulation. The detection of vinculin was used as loading control for sample normalization. Complete Western Blot acquisitions from E-selectin, P-selectin, VCAM-1 and vinculin is depicted in the Supplementary Figure S1.

CAEC were stimulated with *Av*Ag (PBS-soluble L3 protein extract) and samples were analysed via a kinetic approach (3, 6, 12 and 24 h after stimulation). To monitor endothelial cell activation, the expression of the adhesion molecules P-selectin, E-selectin, VCAM-1 and ICAM-1 was assessed. To achieve this, qRT-PCR and Western blotting were performed to quantify mRNA abundance and to estimate protein expression, respectively. In this context, ICAM-1 was not estimated, considering that the commercially available primary antibodies did not work reliably for the canine samples. In all cases, results showed highly variable responses depending on individual canine endothelial cell donors/CAEC isolates (Figure 4 and Figure 5). Referring to mRNA expression, we observed one “non-respondent” CAEC (isolate 2) during the experiments, which hampered significance (based on animal trial restrictions, we could not enhance the number of CAEC isolates), but which might also reflect the true in vivo situation in individual dogs suffering canine angiostrongylosis. Nevertheless, the other two isolates (isolates 1 and 3) reacted upon *Av*Ag stimulation, thereby indicating endothelial cell activation, but followed different reaction patterns (Figure 4). Interestingly, *Av*Ag-stimulated CAEC isolate 1 showed a rather high level of E-selectin, P-selectin and VCAM-1 gene transcripts (Figure 4) when compared to the other isolates. At 12 h post incubation, a peak of E-selectin mRNA expression was observed (Figure 4A) in 2/3 isolates. For P-selectin, a more or less constantly high mRNA abundance was detected but only in isolate 1, whereas ICAM-1 seemed to increase at 6 h post stimulation in 2/3 isolates (Figure 4C). A further increase in 1/3 isolates was observed for ICAM-1 24 h post incubation (Figure 4C). VCAM-1 mRNA expression showed the most variable results with no coincidence between the three isolates (Figure 4D). For positive controls, CAEC isolates were stimulated with TNF-α for 24 h. Given that these data revealed a high variability between CAEC donors for all adhesion molecules tested, this might also indicate an extraordinary individual reactiveness of canine endothelial cell isolates, even in response to such a potent stimulant (Figure 4).

Considering protein expression, adhesion molecule-related results in principle mirrored the reactivities observed in mRNA-based experiments, and revealed highly variable inter-animal responses (Figure 5). Overall, the distinct adhesion molecule regulation in each CAEC isolate reflected an *Av*Ag-driven endothelial cell activation. E-selectin expression increased at 6 h post incubation in 2/3 isolates. In 1/3 isolates this peak already occurred at 3 h post incubation (or earlier). In 1/3 isolates a ≥ 3-fold increase of E-selectin expression when compared to control was observed at 24 h of incubation (Figure 5B). P-selectin protein expression also showed a peak of around two-fold at 6 h post *Av*Ag stimulation in 2/3 isolates, decreasing at 12 h for 2/3 isolates and with a further increase at 24 h (Figure 5C). The latter increase was more pronounced in 1/3 isolates with a four-fold up-regulation of P-selectin protein expression compared to control conditions (Figure 5C). Finally, in line with mRNA expression data, VCAM-1 abundance showed the most variable results in stimulated CAEC (Figure 5D). Thus, a first peak of expression was observed at 3 h post incubation in 1/3 isolates and a second, less pronounced peak at 12 h. In 1/3 isolates the peak occurred at 6 h, but at a much lower level than in isolate 2. Overall, we observed an increased expression of E-selectin, P-selectin and VCAM-1 at protein level in the three different CAEC isolates stimulated with soluble *Av*Ag, which indeed reflected an antigen-driven activation of these endothelial cells. However, regarding the quantity or kinetics of protein expression, the pattern remains inconclusive due to high inter-isolate variations.

## 4. Discussion

After ingestion by the definitive host, *A. vasorum* L3 larval stages must first migrate through the intestinal wall to reach mesenteric lymph nodules where they moult into L4 within the first four days post infection (p.i.), and then invade lymph/blood vessels, and later on the pulmonary arteries, ventricle, atrium and auricle of the right section of heart [1,5,53,54]. Following the definitive host infection, *A. vasorum* are constantly exposed to the definitive host innate immune environment, mainly composed of cell barriers [e.g., digestive mucosa (epithelial cells), endothelium], cells of the innate immune system (e.g., PMN), complementary factors, antimicrobial peptides, cytokines/chemokines, among others. However, studies on early innate canine immune reactions against *A. vasorum* stages are scarce. Given that PMN infiltration is common in *A. vasorum*-infected dogs [54,55,56] and that NETs formation was recently reported as an effective PMN-derived defense mechanism against nematode stages [30,32,37,39,40], we here analysed *A. vasorum* L3-induced NETosis as part of early innate immune responses in the canine system. Considering that *A. vasorum* circulating antigens are in direct contact with highly immunoreactive host endothelial cells of vessels in vivo, vasculitis, perivasculitis and thrombosis are consistently reported for canine angiostrongylosis [54,56]. Therefore, we further studied canine endothelium-derived responses to soluble *Av*Ag. Overall, we here provide first evidence of *A. vasorum*-induced formation of different NET phenotypes, NET-mediated larval entanglement and activation of canine endothelial cells (based on pro-inflammatory adhesion molecule up-regulation).

NETs are composed of decondensed chromatin decorated with histones and granular components, such as calprotein, NE, MPO, cathepsin G, proteinase 3, lactoferrin, LL37, pentraxin and gelatinase, among others [21,57]. Consistently, typical NET-associated components were here confirmed for *A. vasorum* L3-induced NETs by demonstrating co-localization of NE and histones on DNA-rich extracellular fibres being released from canine PMN. In line with other reports on parasites triggering NETosis [8,27,30,58], the induction of different NET phenotypes was here observed. In principle, all types (*spr*NETs, *diff*NETs, *agg*NETs) were detected upon contact of canine PMN with *A. vasorum* L3; however, the most abundant were *spr*NETs, which is consistent with findings on *D. immitis*-triggered NETosis [30]. Referring to functionality of different NET phenotypes, *agg*NETs are reported to have anti-inflammatory properties via sequestration and detoxification of global histones and proteolysis of pro-inflammatory chemokines and cytokines [29,59,60], whilst *spr*NETs and *diff*NETs exhibit pro-inflammatory effects in the early phase of innate response [61]. In the current study, *A. vasorum*-triggered NET formation was quantified via NAE-based estimations using DANA, thereby reflecting early reactions during the NETotic process. This technique was recently demonstrated as useful and reliable in identifying early NETotic cells during parasite encounter [62]. In the current study, an increase in NAE in *A. vasorum* L3-confronted canine PMN confirmed the parasite-triggered induction of NETotic cells and these data corresponded well to observer-based microscopic observations of NETs. Worth noting, DANA has already been successfully performed before in human, murine and bovine systems [25,27,63,64], with the current report representing the first application for canine PMN.

Studies on helminth-triggered NETosis documented the efficiency and strength of delicate extracellular fibers in capturing these large-sized parasites [8,32,37,39,62,65]. Strikingly, mammalian PMN seem able to recognize or sense large-sized parasites [66] and to rapidly cast NETs in response, in order to immobilize these pathogens [67]. On a mechanistic level, NE is slowly released into the cytosol after PMN encounter a pathogen via a route that does not involve membrane fusion, thereby facilitating NE translocation into the nucleus, which finally results in chromatin decondensation and NETs release [66]. Considering the high motility of *A. vasorum* L3, attachment of these larvae onto coverslips is revealed as problematic, even when applying adhesion-promoting compounds, such as poly-_L_-lysine [8,32,62]. Irrespective of coverslip treatments, vital *A. vasorum* L3 were consistently moving away from PMN to areas of coverslips where PMN were less abundant. Despite these efforts of escape, PMN successfully captured several larvae as visualized by SEM; nevertheless, future studies are required to confirm the importance of this observation in vivo. Current observations mainly indicated suicidal NETs formation, which corresponds well to previous NET-related reports on motile protozoan and metazoan parasites [8,27,30,38,62].

Given that PMN are highly present in the blood circulation, complex interactions between this innate immune cell type and activated endothelial cells of blood and lymphatic vessels have been described and also play a fundamental role in the pathogenesis of various parasite infections [42,68,69,70]. Thus, changes in the permeability of endothelium upon inflammation or parasite infection are common [42,71]. Analyses of the expression of typical adhesion molecules serve as indicator of endothelial cell activation. Therefore, we here monitored expression profiles of P-selectin, E-selectin, VCAM-1 and ICAM-1 in parasite antigen-stimulated canine primary endothelial cells. Even though adhesion molecule-related reactions proved highly variable between time points and isolates, they reflected *Av*Ag-driven endothelial activation, thus forming part of early host innate immune response against *A. vasorum*. This variation in endothelium-derived pro-inflammatory reactions were also confirmed between the different CAEC donors used. Therefore, individual variations concerning host innate reactions might be linked to different clinical manifestations during canine angiostrongylosis as previously reported [18]. In line with the current data, several studies on canine angiostrongylosis reported on pathological findings which are in the long term necessarily linked to endothelial cell activation. Thus, formation and endothelial adhesion of antigen-antibody-complexes, thrombus formation and vessel inflammation lead to altered endothelial physiology and integrity [54,55,56]. An indirect evidence of endothelial alteration in canine angiostrongylosis comes from vWF-related findings. vWF is considered as a typical marker of activated endothelial cells. It captures circulating platelets to the site of vascular injury and mediates subsequent platelet activation and aggregation [72,73]. Interestingly, vWF serum levels were consistently found elevated in naturally *A. vasorum*-infected dogs [12,74], thereby most likely reflecting endothelial cell activation. Multimers of vWF, released from activated endothelium, were recorded as spontaneously recruiting excessive circulating platelets and PMN, thereby promoting intravascular thrombosis [72], which is commonly observed in severe cases of canine angiostrongylosis [54,56]. Of note, the metalloprotease ADAMTS13, which is also present in PMN granules [75], specifically cleaves vWF-A2 domains to regulate the size and activity of vWF multimers [76,77], thereby hampering thrombus formation [73,78]. Linking these events, several studies showed that NETs are also involved in venous and arterial thrombus formation [73]. Moreover, NETs directly interact via electrostatic forces and by DNA or H2A with endothelium [42,76] and eventually directly affect endothelial physiology. Taking into account that both vWF and NETs own pro-thrombotic and pro-inflammatory properties, it seems plausible to speculate that interactions between *A. vasorum*-induced NETs and vWF might promote the development of coagulopathies and bleeding disorders in clinical canine angiostrongylosis [12]. Moreover, future studies are necessary to link both processes in order to better understand the whole complex cascade of *A. vasorum*-induced coagulopathies and bleeding disorders in vivo.

## 5. Conclusions

Overall, we here demonstrated for the first time that exposure of primary canine endothelial cells to soluble *Av*Ag resulted in pro-inflammatory activation as part of early host innate immune response against axenic *A. vasorum* L3. Finally, *A. vasorum* L3 was able to strongly induce NETosis in canine PMN and *spr*NETs being the most abundant phenotype observed.

## Figures and Tables

**Table 1:** Sequences of canine probes and primers used for qRT-PCR experiments

	Primers	
Target	Forward/Reverse	Tm
*Canis lupus* E-selectin	5′-TGGCTTCAGAGGTCTCAGGT-3′ 5′-TCAAAGCACTGCACTCAACC-3′	60 °C
*Canis lupus* P-selectin	5′-CAAAAAGCCTCTCACCGAAG-3′ 5′-ATGCATTCTCCTTGCTTGCT-3′	60 °C
*Canis lupus* ICAM-1	5′-CAGGGTTGCCAGGTACAGTT-3′ 5′-AGTATGGGCTCAGTGGGTTG-3′	60 °C
*Canis lupus* VCAM-1	5′-TCCATCGTGGAGGAAGGTAG-3′ 5′-CAGCCTGGTTAATCCCTTCA-3′	60 °C
*Canis lupus* RPL32	5′-CCTCAGACCTCTGGTGAAGC-3′ 5′-TCAAGCTCCTTGACGTTGTG-3′	60 °C
*Canis lupus* RPS19	5′-TGTCAAGGCTACCTCGGAGT-3′ 5′-GCCTTCAGCCTCCTTCTTCT-3′	60 °C
*Canis lupus* HPRT	5′-AAGCTTGCTGGTGAAAAGGA-3′ 5′-CAATGGGACTCCAGATGCTT-3′	60 °C
Probes		
*Canis lupus* E-selectin	5′-TTTGTCAGCTGTGACAAGGG-3′	60 °C
*Canis lupus* P-selectin	5′-GCTATACAGCCTCCTGCCAG-3′	60 °C
*Canis lupus* ICAM-1	5′-CATTGGCTAAGCTGCTTTCC-3′	60 °C
*Canis lupus* VCAM-1	5′-GAGCAGGCGGCTAAGTAATG-3′	60 °C
*Canis lupus* RPL32	5′-GGCACCAGTCAGACCGATAT-3′	60 °C
*Canis lupus* RPS19	5′-CAGTCACCCAGCAGATTGTG-3′	60 °C
*Canis lupus* HPRT	5′-CCCCTCGAAGTGTTGGCTAT-3′	60 °C

## Data Availability

The raw data supporting the conclusions of this article will be made available by the authors, without undue reservation.

## Supplementary Materials

The following are available online at https://www.mdpi.com/article/10.3390/biology10050427/s1, Figure S1: Complete Western Blot acquisitions from E-Selectin, P-Selectin, VCAM-1 and Vinculin.
