# Supplementary figures and images for: Canine Angiostrongylus vasorum-Induced Early Innate Immune Reactions Based on NETs Formation and Canine Vascular Endothelial Cell Activation In Vitro
Source: Biology (Basel). 2021 May 12;10(5):427. doi: 10.3390/biology10050427 (PMC8151090; doi:10.3390/biology10050427)

CAEC E-Selectin

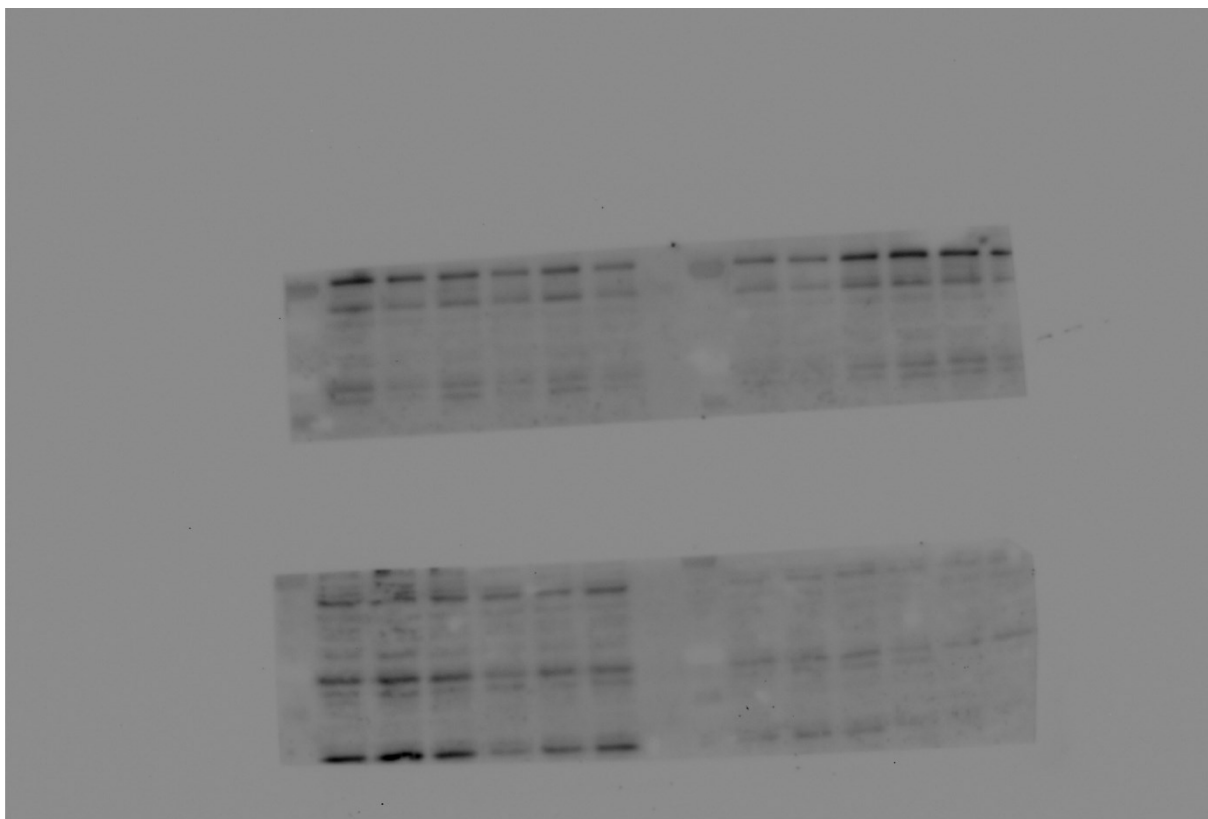

CAEC P-Selectin

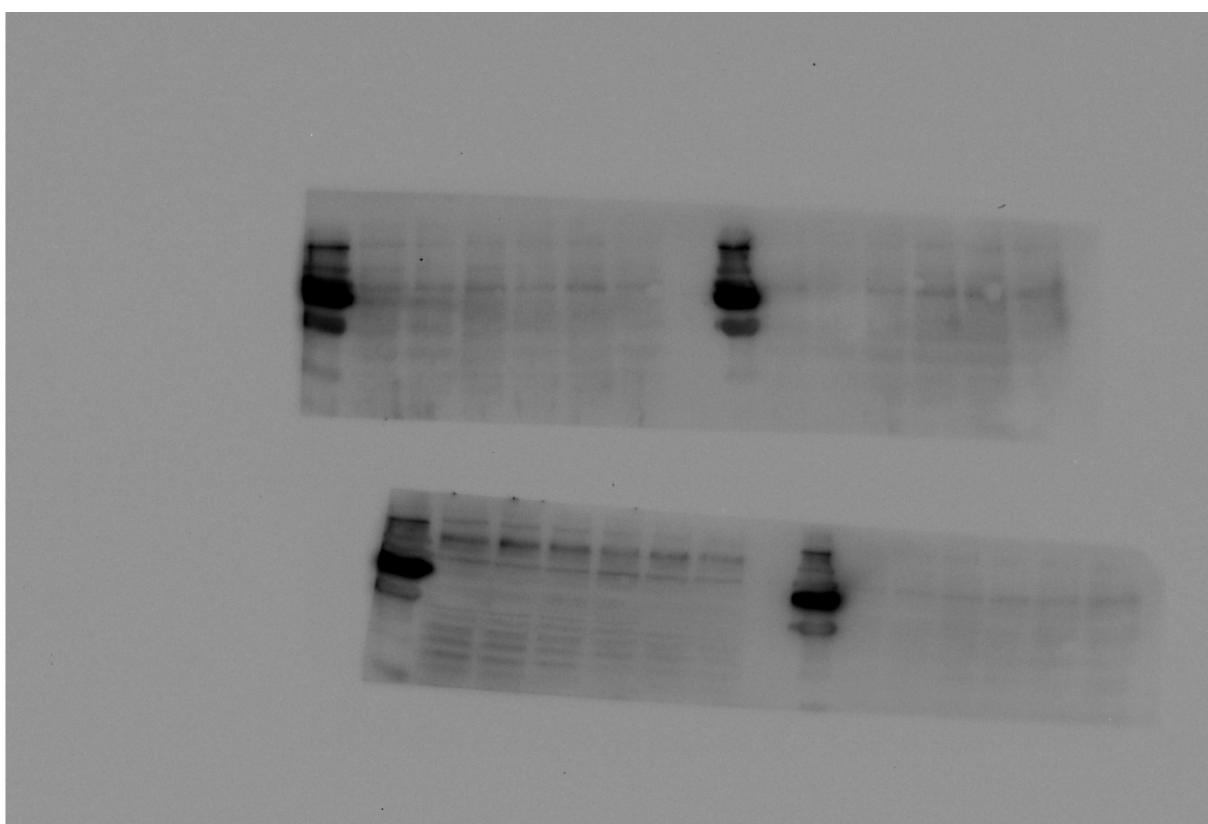

CAEC VCAM-1

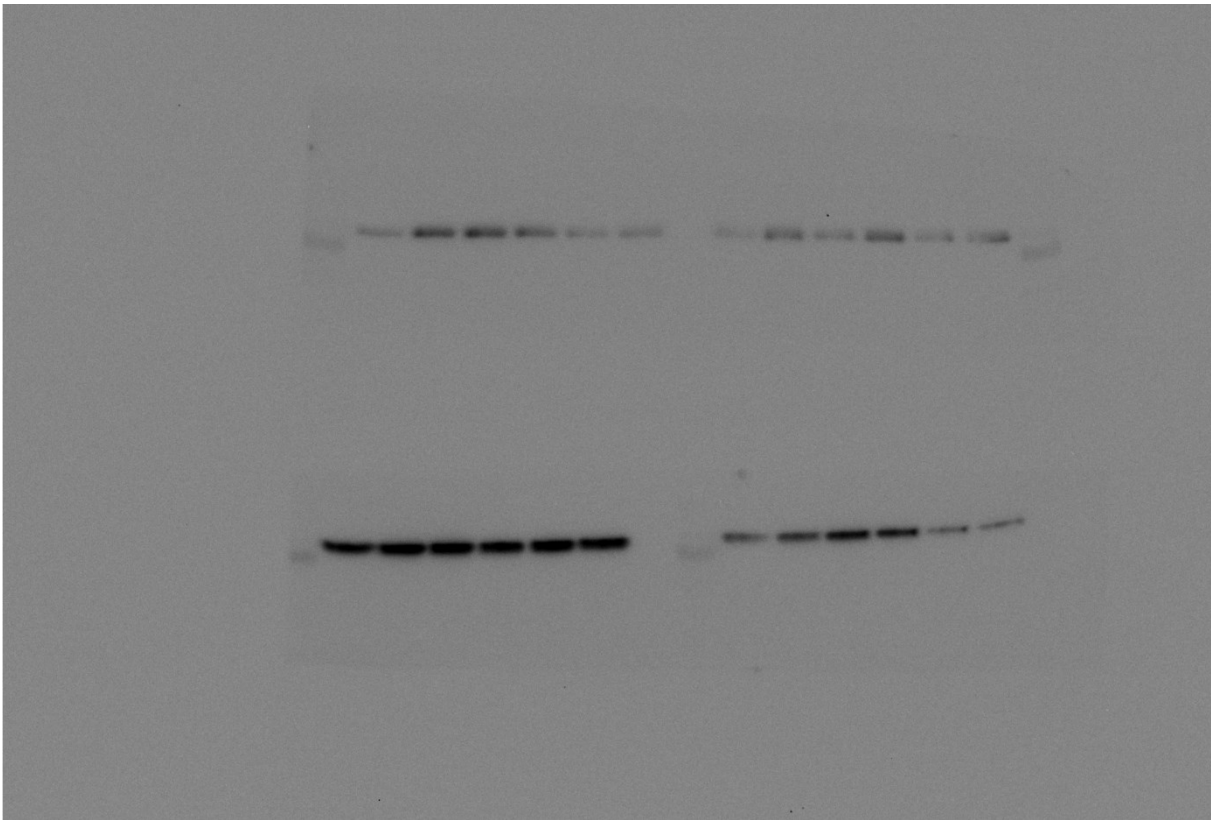

CAEC Vinculin

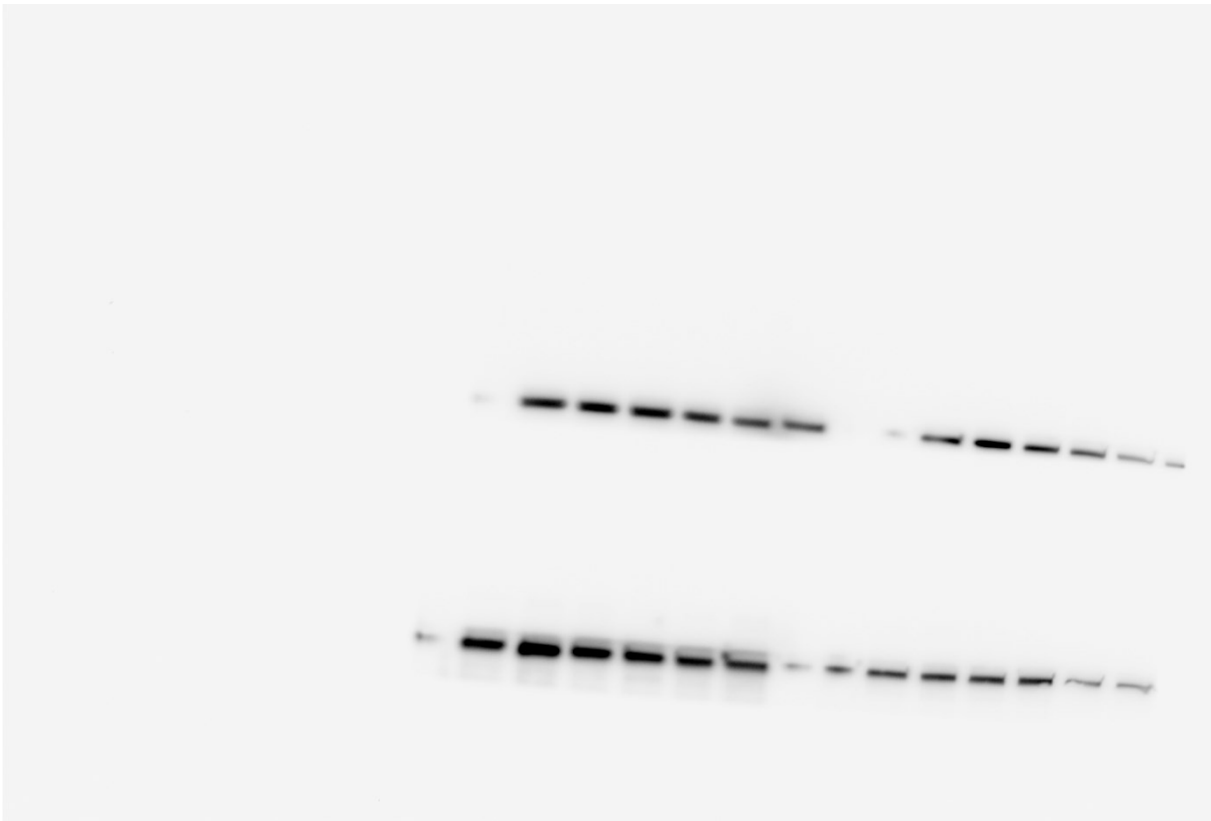

Supplement: Supplementary file 1 [file biology-10-00427-s001.zip › biology-1182151-supplementary.pdf]
